# Willingness to Share Smartwatch-Generated Health Data Among a Sample of Adults in Riyadh, Saudi Arabia

**DOI:** 10.3390/healthcare14172838

**Published:** 2026-09-03

**Authors:** Raniah N. Aldekhyyel, Leena M. Shagrani, Shoug M. Albattah, Basmah A. Alghamdi, Adwaa A. Alsalman, Shadin K. Alabbas, Haya E. Alkhlaiwi

**Affiliations:** 1 Medical Informatics and e-Learning Unit, Medical Education Department, College of Medicine, King Saud University, Riyadh 12372, Saudi Arabia; 2 College of Medicine, King Saud University, Riyadh 12372, Saudi Arabia

**Keywords:** smartwatches, wearable devices, health-generated data, data sharing, patient engagement, digital health

## Abstract

**Background/Objectives:** Wearable devices, such as smartwatches, represent an emerging digital health tool that can enhance patient engagement, support patient-provider communication, and enable more personalized, patient-centered care. Understanding public willingness to share health-generated data with healthcare providers is essential for informing digital health implementation strategies that prioritize patient trust and active participation in the care process. This study aimed to address this gap by assessing the willingness of a sample of smartwatch users in Riyadh to share their health-generated data and identifying factors associated with this willingness. **Methods:** A cross-sectional study was conducted using venue-based convenience sampling at a large public venue in Riyadh, Saudi Arabia. Data were collected using an electronic self-administered questionnaire. Descriptive statistics summarized participant characteristics, while bivariate analyses and multivariable logistic regression were used to examine factors associated with willingness to share health-generated data. **Results:** Among the 391 participants, 168 reported owning a smartwatch and comprised the population for the primary analysis of willingness to share health-generated data. Among smartwatch owners (n = 168), 104 (62%; 95% CI: 54–69%) expressed willingness to share their health-generated data. Among those willing to share, healthcare providers were the most preferred recipients (95%, 99/104), followed by family members and friends (63%, 66/104). Most demographic, socioeconomic, health-related, and behavioral characteristics were not significantly associated with willingness to share; however, in bivariate analysis, previous use of online support groups was significantly associated with greater willingness to share health-generated data (OR 5.00; 95% CI: 1.66–15.10; *p* = 0.002). None of the predictors included in the multivariable model was independently associated with willingness to share. **Conclusions:** Most smartwatch users were willing to share their health-generated data with healthcare providers, reflecting positive public attitudes toward integrating this data into healthcare systems. These findings support Saudi Arabia’s digital health transformation initiatives, while highlighting the need to address privacy, trust, and data governance to enable successful implementation.

## 1. Introduction

The rapid growth of wearable health technologies [1], particularly smartwatches, has transformed how individuals monitor and engage with their own health [2], creating new opportunities for patient engagement and communication between patients and healthcare providers [3,4]. These devices fall under the broader category of wearable technology [5], referring to electronic devices equipped with sensors that continuously monitor physiological or behavioral data such as heart rate, physical activity, and sleep patterns [6]. Owing to their computing and physiological monitoring capabilities, smartwatches have evolved into important healthcare tools that support diagnosis [7], clinical decision-making [8], remote patient monitoring [3], and preventive and personalized care [9]. Marking a broader shift from consumer electronics toward clinically relevant technology, these tools have also been integrated into modern digital health ecosystems for cardiovascular medicine [10] and chronic disease management [11,12,13,14]. As smartwatches become increasingly integrated into everyday life [15], understanding whether users are willing to share the health data these tools generate with healthcare providers has become a critical question for advancing patient-centered [16], digital health implementation worldwide [17,18,19,20].

International research demonstrates high willingness among patients to share health data generated from smartwatches, especially with healthcare providers. Rising et al. [21] found that around 82% of U.S. adults were willing to share health data with healthcare providers, and around 70% were willing to share with family or friends, with behavioral factors such as technology use, physical activity, and trust in healthcare professionals emerging as stronger predictors of willingness than demographic characteristics [21]. However, willingness to share health data does not always translate into actual behavior. Chandrasekaran et al.’s [22] research showed that while 78% of participants expressed willingness to share health-generated data, only 26% had actually done so, with privacy concerns, uncertainty regarding data use, and system integration challenges cited as key barriers [22]. Trust in healthcare providers and in the broader healthcare system may similarly shape willingness. Individuals are more likely to share data when they are assured it will be used securely [17]. Effective data sharing also depends on governance structures capable of ensuring data privacy and security [23,24,25]. Additional findings from Zhang et al. [26] suggest that sharing health-generated data from smartwatches can strengthen confidence in digital health tools and improve communication with healthcare professionals, particularly among older adults [26]. Overall, the literature suggests that willingness to share health-generated data from smartwatches is influenced more by behavioral and psychological factors, such as trust, technology use, and health self-efficacy than by demographic factors like age or gender [17,19,20,21,22,26].

Although fewer studies exist in the Middle East, available Saudi research offers some insight into wearable technology adoption. A study conducted by Alaskari et al. [27], indicated that 94% of participants were aware of smartwatch technologies, with age, education, and income significantly influencing adoption [27]. Other studies using technology acceptance models identified perceived usefulness, device design, and health information support as key drivers of wearable adoption in Saudi Arabia [28]. However, these studies focus on adoption rather than willingness to share data, and psychological and behavioral factors such as trust in healthcare providers, technology literacy, and self-efficacy remain under-explored in the Saudi context.

This gap is particularly significant given Saudi Arabia’s ongoing Health Sector Transformation Program and E-health initiative [29], which is part of the country’s Vision 2030 [30]. The initiative aims to improve healthcare efficiency, integration, and quality through advanced information technologies, with the goal of establishing a unified electronic medical record system connecting healthcare facilities nationwide [31]. Achieving this goal will require robust technical safeguards to protect the integrity and confidentiality of medical records, an area addressed in the broader technical literature on securing health information systems [32]. A unified electronic medical record system enables continuous access to patient data, supports evidence-based clinical decision-making, and enhances healthcare service effectiveness [33]. Examining willingness to share health data aligns directly with Vision 2030’s Health Sector Transformation Program [29], which prioritizes digital health technologies as a means of improving population health outcomes and strengthening the relationship between patients and the healthcare system [33].

Despite growing international evidence on willingness to share health-generated data from smartwatches, comparable research remains scarce in Saudi Arabia. As a result, the demographic, socioeconomic, and health-related factors that may shape this willingness remain largely unexplored. This gap limits the ability of policymakers and healthcare systems to design digital health strategies that reflect the preferences, concerns, and trust-related needs of the local population.

This study aimed to address this gap by assessing the willingness of a sample of smartwatch users in Riyadh to share their health-generated data and identifying factors associated with this willingness. In doing so, this study seeks to inform patient-centered digital health strategies aligned with Saudi Arabia’s national 2030 Vision in transforming healthcare.

## 2. Materials and Methods

This study employed a cross-sectional design among a sample of Saudi adults in Riyadh to assess the willingness of smartwatch owners to share their health-generated data. Data were collected using a structured, self-administered questionnaire distributed at a large public venue, and analyzed using descriptive, bivariate, and multivariable statistical approaches. The following subsections describe the study setting and sampling approach, questionnaire development and validation, recruitment and data collection procedures, and statistical analysis.

### 2.1. Study Design, Setting, and Sampling Technique

This study employed an observational, analytical, cross-sectional design. It was conducted in Riyadh, Kingdom of Saudi Arabia, between January and April 2026. The target population consisted of a sample of Saudi citizens aged 18 years and older residing in or visiting Riyadh during the time of the study. The study focused specifically on Saudi citizens, given the limited existing research examining this population’s willingness to share digital health data. Individuals younger than 18 years and non-Saudi residents were excluded from participation. Venue-based convenience sampling, a non-probability sampling method, was used due to its practicality and efficiency in accessing the target population within the available time and resources.

The sample size was calculated using the formula for estimating a population proportion, with a 95% confidence level (Z = 1.96), *p* = 0.5 to ensure maximum variability, and d = 0.05 as the margin of error. The minimum calculated sample size was approximately 384 participants. This calculation was based on the overall study population. No separate a priori sample-size calculation was performed specifically for the subgroup of smartwatch owners included in the primary willingness-to-share analysis.

### 2.2. Questionnaire Development and Validation

The study utilized a standardized online questionnaire adapted from the Health Information National Trends Survey (HINTS), an open-access instrument developed by the National Cancer Institute [34]. The research team thoroughly reviewed the full HINTS 5, Cycle 3 instrument [35] and identified Sections A “Looking for Health Information”, B “Using the Internet to Find Information”, F “Your Overall Health”, and H “Physical Activity and Exercise” as relevant to the study’s objectives. Within these sections, individual items were then selected based on their relevance to the study’s aims and the characteristics of the target population; accordingly, not all items from the selected sections were included in the final questionnaire.

To ensure validity, the selected items were first reviewed by five content experts to confirm their relevance to the study objectives, clarity, and cultural appropriateness. The questionnaire was then translated into Arabic by an independent bilingual translator. Established guidelines for the cross-cultural adaptation of self-report measures typically recommend forward translation combined with independent back-translation and reconciliation by an expert committee [36,37]. However, in this study, only forward translation was performed, and back-translation was not conducted due to time constraints. To ensure semantic and conceptual equivalence in the absence of formal back-translation, the same five content experts subsequently reviewed the questionnaire a second time, comparing the Arabic and original English versions. Experts provided qualitative feedback on translation accuracy, item clarity, and cultural appropriateness, rather than structured numeric relevance ratings from which a formal Content Validity Index could be calculated. The questionnaire was revised based on the experts’ feedback wherever discrepancies were identified. Following this review, the questionnaire was assessed by 10 smartwatch users to evaluate the clarity and comprehensibility of the items from the perspective of the target population. Based on their feedback, minor revisions were made to the order of the questions.

The final questionnaire consisted of four sections: (1) demographic characteristics, including age, gender, region of residence, educational level, income, and financial status; (2) health-related characteristics, including general health status, chronic illnesses, physical activity, and trust in healthcare providers; (3) digital health behaviors, including internet use and smartwatch utilization; and (4) willingness to share health data with healthcare providers and/or family members. Sections (2), (3), and (4) were adopted from HINTS Sections A, B, F, and H, as previously described. Items were presented in yes/no, Likert-scale, and multiple-choice formats. The full questionnaire is provided in Appendix A. For the item “How confident are you in your ability to take good care of your health?”, responses were grouped into three categories for analysis: “completely confident” and “very confident” were combined into a high confidence category; “somewhat confident” was classified as moderate confidence; and “not at all confident” and “a little confident” were combined into a low confidence category. Trust in health information from healthcare providers and from family and friends was assessed using the corresponding questionnaire items asking participants how much they trusted health or medical information from each source. Responses were categorized into three levels for analysis: “not at all” and “a little” were combined as low trust, “some” was categorized as moderate trust, and “a lot” was categorized as high trust. The two lowest response categories were combined because of the small number of responses in the “not at all” category.

For analyses related specifically to smartwatch use and willingness to share health-generated data (Section (4) of the questionnaire), only smartwatch owners were included, as these questions were not applicable to non-owners.

### 2.3. Recruitment and Data Collection Procedures

Participants were recruited at Roshn Front, one of Riyadh’s major public venues, which hosts events and attracts visitors from across the country throughout the year, in addition to local residents. Venue-based recruitment at high-traffic public locations is a well-established approach for efficiently reaching broad, heterogeneous samples of the general public [38], and has been similarly applied in Saudi public health research conducted at shopping malls and other public venues in Riyadh and Jeddah [39,40,41].

Eligible participants were approached by members of the research team and invited to participate voluntarily. After participants agreed to be part of the study, and prior to accessing the survey questions, they were required to provide consent electronically through Google Forms. Participants completed the questionnaire electronically and privately using Google Forms, either through a handheld tablet provided by the research team or by scanning a barcode link with their personal smartphones. The research team remained available during questionnaire completion to provide clarification and technical assistance when needed, without influencing participants’ responses. Data were collected anonymously, and no personally identifiable information was recorded. No incentives were given to participants.

### 2.4. Statistical Analysis

Data were analyzed using IBM SPSS Statistics (Version 21) (IBM Corp., Armonk, NY, USA). Descriptive statistics, including frequencies and percentages, were used to summarize categorical variables. For the item “How confident are you in your ability to take good care of your health?”, responses were grouped into three categories for analysis: “completely confident” and “very confident” were combined into a high confidence category; “somewhat confident” was classified as moderate confidence; and “not at all confident” and “a little confident” were combined into a low confidence category.

Bivariate associations between participant characteristics and willingness to share health-generated data were assessed using Pearson’s chi-square test or the Fisher–Freeman–Halton exact test when any expected cell count was below five. Unadjusted odds ratios with 95% confidence intervals accompany every comparison. Predictor variables included in the multivariable logistic regression models were selected based on their clinical and theoretical relevance to the respective outcomes, rather than on statistical significance in the bivariate analyses.

Two multivariable logistic regression models were fitted. The first modelled willingness to share health-generated data among smartwatch owners (n = 168) and the second modelled smartwatch ownership across the full sample (n = 391). Model performance was assessed using the likelihood-ratio test, McFadden and Nagelkerke pseudo-R^2^, the area under the receiver operating characteristic curve, and the Hosmer–Lemeshow goodness-of-fit test; multicollinearity was assessed using variance inflation factors.

## 3. Results

### 3.1. Participants’ Sociodemographic Characteristics

**Figure 1:**
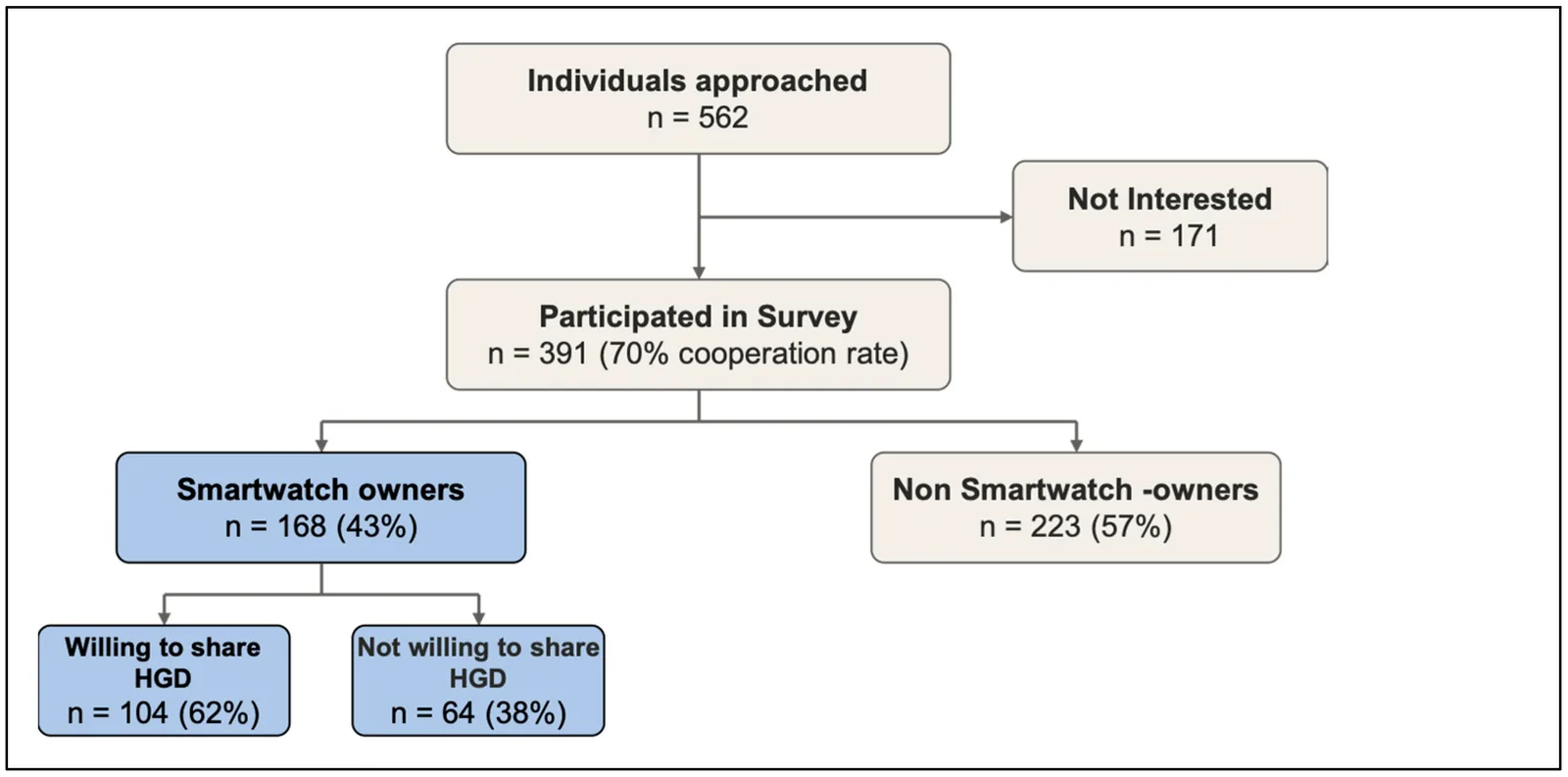
Participant selection process. HGD: health-generated data.

A total of 562 individuals were approached during venue-based recruitment, of whom 391 agreed to participate and complete the questionnaire (cooperation rate: 70%). Among the 391 who participated in the study, 168 (43%) reported owning a smartwatch, while the remaining 223 (57%) did not (Figure 1).

The majority of participants were female (58%) and aged 18 to 34 years (44%). Most were from the central region (91%) and had a college degree or above (73%). Over half reported a monthly income between 10,000 and 30,000 SAR (56%). Most participants described their financial situation as either managing with their existing income (47%) or living comfortably (43%) (Table 1).

### 3.2. Health Status and Health Behavioral Characteristics

In terms of self-rated health status, 60% of participants described their health as good, while 4% reported poor health. Confidence in managing one’s own health was high among 54% of participants and low among 7%. Chronic health conditions were relatively uncommon in this sample (77%), with depression or anxiety reported by 11% of participants and diabetes reported by 8%. For physical activity, 68% of participants reported activity levels below 150 min per week. Trust in sources of health information varied by domain: 52% of participants reported moderate trust in family and friends, and 49% reported high trust in healthcare providers (Table 2).

### 3.3. Digital Health Behaviors

**Figure 2:**
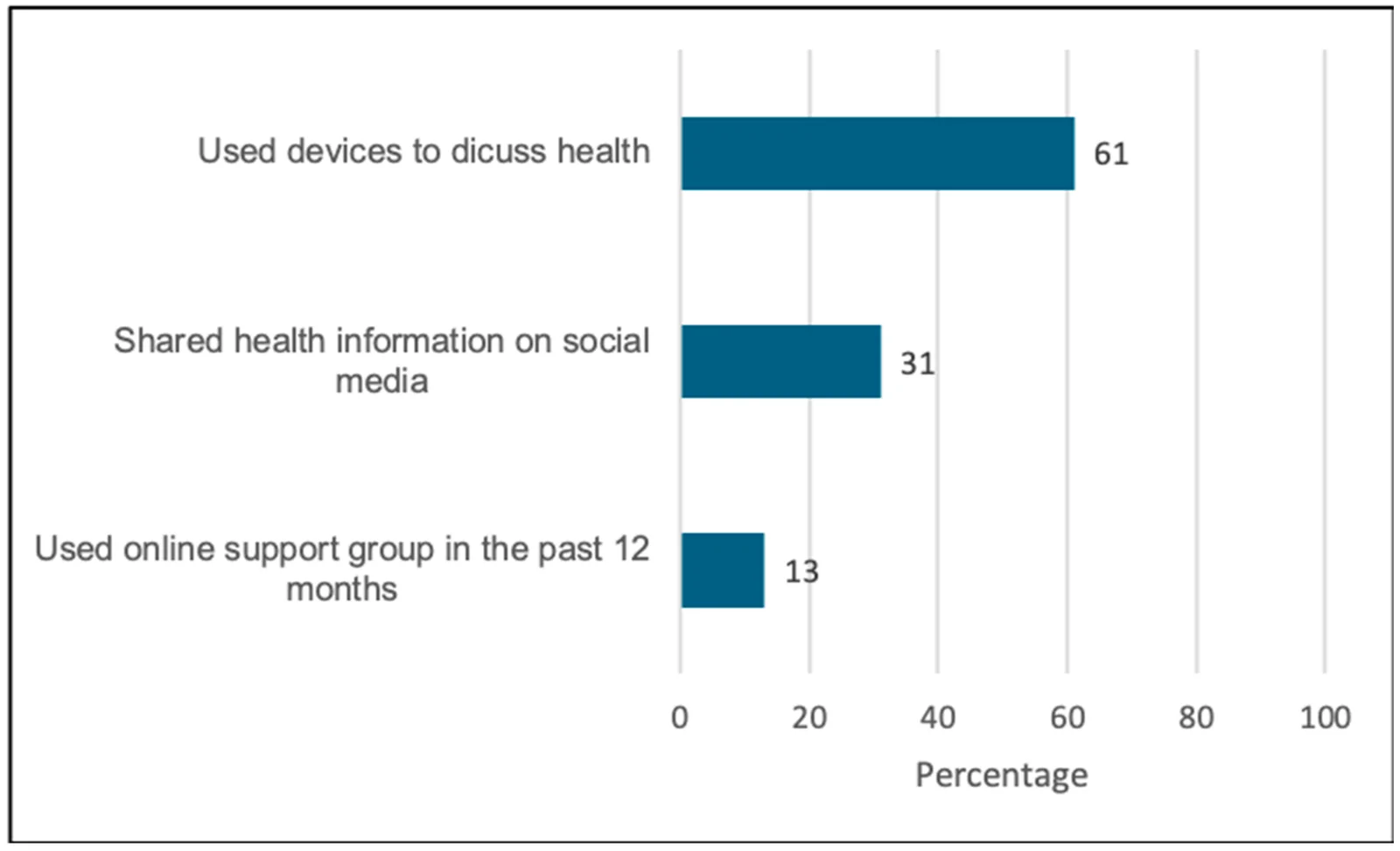
Digital health behaviors (n = 391).

Use of digital health tools was common among participants. Most respondents reported using devices to discuss health (61%), and 31% shared health-related information on social media. However, only 13% had used online support groups (Figure 2).

### 3.4. Smartwatch Owners’ Usage Patterns and Data Sharing

Questions related specifically to smartwatch use and willingness to share health-generated data were applicable only to smartwatch owners. A total of 168 participants (43%) reported owning a smartwatch. Among smartwatch users (n = 168), usage patterns varied. While 51% of participants reported high-frequency use (≥3 times per week), a notable proportion (28%) reported no use within the past month. Of the 168 smartwatch owners, 104 (62%; 95% CI: 54–69%) expressed willingness to share their health-generated data. Among those willing to share (n = 104), there was a strong preference for sharing data with healthcare providers (95%), compared to sharing with family and friends (63%) (Table 3).

### 3.5. Factors Associated with Willingness to Share HGD

In the multivariable model among smartwatch owners (Table 4), none of the six predictors were associated with willingness to share HGD.

Bivariate associations between willingness to share HGD and each participant characteristic are presented in Table 5. No statistically significant associations were observed, except for the use of online support groups. Participants who used online support groups had significantly higher odds of being willing to share HGD than those who did not use such groups (OR, 5.00; 95% CI, 1.66–15.10; χ^2^ *p* = 0.002).

A second model examined predictors of smartwatch ownership across all 391 participants (Table A1). Ownership declined sharply with age, with participants aged 50 years and above having markedly lower odds of ownership than those aged 18 to 34 years (aOR 0.20, 95% CI 0.11–0.39, *p* < 0.001). Participants reporting good (aOR 4.63, 95% CI 1.09–19.65, *p* = 0.038) or excellent (aOR 4.63, 95% CI 1.02–20.95, *p* = 0.046) general health had higher odds of ownership than those reporting poor health, although these intervals are wide because only 17 participants rated their health as poor. Reporting any chronic condition was also associated with higher odds of ownership (aOR 1.88, 95% CI 1.03–3.42, *p* = 0.039).

## 4. Discussion

This study assessed willingness among smartwatch owners to share health-generated data and examined demographic, socioeconomic, health-related, behavioral, and digital health factors potentially associated with this willingness. Among smartwatch owners, more than half of the participants expressed willingness to share their health-generated data. Among those willing to share, healthcare providers were the most preferred recipients, which may indicate a generally positive receptiveness toward the clinical use of health-generated data. Most participant characteristics were not significantly associated with willingness to share; however, previous use of online support groups was significantly associated with greater willingness in the bivariate analysis. In the multivariable analysis, none of the six included predictors was independently associated with willingness to share.

Smartwatch ownership was reported by less than half of the overall sample, and patterns of use among owners varied considerably. While approximately half of smartwatch owners reported frequent use, a notable proportion had not used their device during the previous month. This suggests that ownership does not necessarily translate into consistent engagement with wearable technology, an important consideration for the broader digital health data ecosystem [9]. Even among individuals with access to wearable technologies, inconsistent use may limit the volume and continuity of health data generated and consequently reduce its potential clinical utility if integrated into routine care [42,43]. This variation in engagement is also relevant when considered alongside participants’ physical activity patterns. Among the overall sample, more than half of the participants reported engaging in less than 150 min of physical activity per week. Given the cross-sectional design of this study, these findings cannot establish a relationship between smartwatch use and physical activity. Nevertheless, they illustrate that access to digital health technologies should not in itself be assumed to result in sustained health-related behavioral change.

Although wearable devices may facilitate awareness and self-monitoring, meaningful behavioral change may additionally depend on factors such as motivation, environmental support, and health education. This is consistent with evidence suggesting that user engagement with wearable devices is important for improvements in physical activity and reductions in sedentary behavior [44]. Future implementation efforts may therefore need to consider not only willingness to share data but also strategies that encourage sustained and meaningful smartwatch use among owners. Despite variability in smartwatch use, willingness to share health-generated data was moderate among participants in the sample, with a particularly strong preference for sharing with healthcare providers rather than family members or friends. This finding is consistent with international research demonstrating generally positive attitudes toward sharing wearable-generated health data with healthcare professionals [21]. Differences in the preference between populations may reflect variation in cultural context, healthcare systems, privacy expectations, or perceptions regarding trust and data use. The strong preference for healthcare providers as recipients may suggest that participants may distinguish between clinical and informal uses of health data [17], and/or perceive healthcare professionals as a trusted source for this information [22,26]. This distinction is relevant to digital health implementation because receptiveness to sharing health data may depend not only on willingness to share health data, but also on who receives it and how it is expected to be used.

Most demographic, socioeconomic, health-related, trust-related, and smartwatch-use characteristics were not significantly associated with willingness to share in the bivariate analyses. However, previous use of online support groups was significantly associated with greater willingness to share health-generated data. Participants who had previously used online support groups had approximately five times the odds of being willing to share compared with those who had not. This finding may suggest that individuals who are already engaged with digital platforms for health-related purposes are more receptive to sharing digitally generated health information. Previous engagement with online health communities may reflect greater familiarity with digital health environments or greater comfort exchanging health-related information electronically [45]. However, this association should be interpreted cautiously given the relatively small number of participants who reported using online support groups and the number of bivariate comparisons conducted.

In the multivariable logistic regression model, smartwatch usage frequency, trust in healthcare providers, age, education level, health confidence, and having a regular healthcare provider were not significantly associated with willingness to share. The trust measure used in this study, consistent with Rising et al. [21], captured trust in healthcare providers as a source of health information, rather than trust in providers’ capacity to securely manage continuously transmitted personal data. The null finding for trust should therefore be interpreted within this specific scope, and should not be read as evidence that trust in data privacy or security more broadly is unrelated to willingness to share. However, the absence of statistically significant associations in the adjusted model should not be interpreted as evidence that these factors have no relationship with willingness. The primary analysis was restricted to the 168 participants who owned smartwatches, and several adjusted estimates had wide confidence intervals, indicating limited precision in estimating potential associations. Studies with larger samples of smartwatch owners may therefore help clarify whether such relationships exist within the Saudi population.

The findings should also be considered in light of the documented gap between stated willingness and actual data-sharing behavior in other populations [22]. This study assessed willingness rather than observed sharing behavior, and a meaningful proportion of smartwatch owners reported inconsistent device use. Therefore, reported willingness should not be interpreted as evidence that participants would necessarily share their data in practice or that wearable devices would provide a continuous stream of clinically usable information. Translating willingness into effective clinical integration will likely depend on several practical considerations. System usability and interoperability with existing health records are particularly significant, since the clinical value of smartwatch-generated data depends not only on patients’ willingness to share it, but on healthcare systems’ technical capacity to receive, integrate, and act on this data within existing clinical workflows. In the Saudi context, this is directly relevant to the E-Health Initiative’s goal of establishing a unified electronic medical record system connecting healthcare facilities nationwide [29]. Without corresponding interoperability standards for wearable-generated data specifically, patient willingness to share such data may not translate into its meaningful use within clinical care [46]. Achieving this integration will also require attention to privacy and data governance frameworks capable of securely managing continuous, sensitive biometric data streams [23], as well as strategies to support sustained patient engagement with wearable devices over time, given the inconsistent usage patterns observed in this sample [43].

This study contributes to the limited literature on wearable technology in Saudi Arabia, which has primarily focused on awareness, adoption, and use rather than willingness to share health-generated data [27,28]. By examining data-sharing willingness specifically among smartwatch owners while also considering patterns of device engagement, the study provides locally relevant evidence for Saudi Arabia’s evolving digital health landscape. The finding that smartwatch owners were willing to share their data, particularly with healthcare providers, suggests a potentially receptive foundation for the integration of patient-generated health data into healthcare. At the same time, the limited number of clearly identified factors associated with willingness and the variability in actual smartwatch use highlight the need for further research into the practical, behavioural, and system-level factors that may facilitate successful implementation. These considerations are directly relevant to the goals of Saudi Arabia’s Vision 2030 Health Sector Transformation Program and E-Health initiatives [29], which increasingly depend on public acceptance and effective integration of digital health technologies [33].

As an additional analysis, factors associated with smartwatch ownership were examined across the full study sample. The findings identified associations between smartwatch ownership and selected demographic and health-related characteristics. Although smartwatch ownership was not the primary focus of the present study, these findings highlight a potentially important distinction between the factors associated with access to or adoption of wearable technology and the factors associated with willingness to share data among individuals who already own such devices. Smartwatch adoption and willingness to share may therefore represent different stages within the broader pathway toward the clinical use of patient-generated health data.

This distinction may provide a useful direction for future research. Studies specifically focused on smartwatch adoption could examine differences between owners and non-owners in greater depth and investigate the demographic, socioeconomic, health-related, and behavioural factors that influence access to and adoption of wearable technology. Such research could complement studies of data-sharing willingness by examining the earlier stage of the pathway, such as who adopts these technologies. Future research should also directly assess factors such as privacy concerns, perceived data security, and data governance, which were not measured in the present study but may help explain willingness to share wearable-generated health data, as well as the practical barriers to clinical integration, including system usability and interoperability with existing health records. Together, these areas of investigation may provide a more complete understanding of the factors influencing progression from access to wearable technology, to continuous engagement, and ultimately to willingness to integrate wearable-generated health information into clinical care.

### Strengths and Limitations

This study is, to our knowledge, among the first to assess willingness to share health-generated data among a sample of Saudi adults owning a smartwatch, addressing a gap in a largely unexamined regional context.

Several limitations should be considered. Data collection took place at a single site using convenience sampling, introducing selection bias and limiting generalizability. The sample was concentrated among younger, highly educated participants from the Central region and restricted to Saudi citizens, and should be interpreted as an initial, exploratory contribution rather than a nationally representative assessment. Willingness to share data was self-reported and may not reflect actual future behavior, and the presence of research team members during questionnaire completion may have introduced social-desirability bias. Several methodological limitations also apply to the questionnaire itself. Selective adoption of HINTS items rather than complete validated subscales may have affected the instrument’s original psychometric properties. Back-translation was not performed due to time constraints, though content experts subsequently compared the Arabic and English versions to identify and resolve discrepancies. A formal Content Validity Index and reliability statistics were not calculated, as expert feedback was qualitative, and the questionnaire consisted of independent items across distinct constructs rather than multi-item scales.

The primary analyses of willingness were restricted to the 168 smartwatch owners for whom these questions applied, reducing statistical power and limiting the ability to detect modest associations. Non-significant findings should therefore not be interpreted as evidence that no meaningful relationships exist. This limited analytic sample also affected the precision of subgroup comparisons, such as the association observed between prior online support-group use and willingness to share. The supplementary analysis of smartwatch ownership should similarly be viewed as preliminary, given the study’s primary focus on willingness to share rather than ownership itself.

Finally, this study did not directly measure privacy concerns, perceived data security, or trust in data stewardship. Cross-national comparisons of willingness to share data should also be interpreted cautiously, given differences in instruments, sampling frames, and study years across studies.

## 5. Conclusions

This study found that a majority of smartwatch owners were willing to share health-generated data, with healthcare providers being the most preferred recipients. Most demographic, socioeconomic, health-related, trust-related, and smartwatch-use characteristics were not significantly associated with willingness to share. However, previous use of online support groups was significantly associated with greater willingness in the bivariate analysis, while none of the six factors examined in the multivariable model was independently associated with willingness. These findings suggest generally positive receptiveness toward the clinical use of health-generated data, while also highlighting the need to better understand the behavioral and system-level factors that influence whether willingness translates into actual data sharing and sustained engagement with wearable technologies. The supplementary analysis of smartwatch ownership further suggests that factors associated with the adoption of smartwatch technology may differ from those associated with willingness to share data among existing owners. Future research examining smartwatch adoption and data-sharing willingness as distinct but interconnected stages may provide a more comprehensive understanding of the pathway toward integrating patient-generated health data into clinical care in Saudi Arabia.

## Figures and Tables

**Table 1:** Participants’ Sociodemographic Characteristics (n = 391).

Variable	Number (n)	Percentage (%)
**Gender**		
Female	225	58
Male	166	42
**Age group**		
18–34 years	171	44
35–49 years	109	28
50+ years	111	28
**Region**		
Central	357	91
Others	34	9
**Education level**		
Low: High-school graduate or less	69	17
Medium: technical/vocational/diploma	38	10
High: College graduates and postgraduates	284	73
**Family monthly income**		
Low: <10,000 SAR	48	12
Medium: 10,000–30,000 SAR	217	56
High: >30,000	126	32
**Financial status**		
Difficult or very difficult	40	10
Managing with existing income	184	47
Living comfortably	167	43

**Table 2:** Health Status and Health Behavioral Characteristics (n = 391).

Variable	Number (n)	Percentage (%)
**General health**		
Poor	17	4
Good	236	60
Excellent	138	36
**Health confidence**		
Low (Not at all + A little)	27	7
Moderate (Somewhat)	151	39
High (Very + Completely)	213	54
**Chronic conditions** ** ^1^**		
Diabetes	30	8
Heart diseases	16	4
Lung diseases	11	3
Depression or anxiety	42	11
None	303	77
**Weekly physical activity duration**		
Less than 150 min per week	267	68
150 min or more per week	124	32
**Regular health provider**		
Yes	97	25
No	294	75
**Trust health information from family/friends**		
Low (Not at all + A little)	137	35
Medium (Some)	204	52
High (A lot)	50	13
**Trust health information from a health provider**		
Low (Not at all + A little)	19	5
Medium (Some)	180	46
High (A lot)	192	49

^1^ Participants could select multiple chronic conditions or select “None” if no listed condition applied; therefore, responses were not mutually exclusive.

**Table 3:** Usage Patterns and Data Sharing Among Smartwatch Owners (n = 168).

Variable	Number (n)	Percentage (%)
**Smartwatch Usage Frequency**		
Non-user (did not use in the past month)	47	28
Low use (≤2 times/week)	36	21
High use (≥3 times/week)	85	51
Willing to Share HGD *	**104**	**62**
Willingness to Share HGD with Healthcare Provider **	99	95
Willingness to Share HGD with Family and Friends **	66	63

* HGD: Health-generated data. ** Percentages calculated among participants who expressed willingness to share HGD (n = 104). Participants could select one or both (healthcare providers, family and friends).

**Table 4:** Multivariable Logistic Regression of Factors Associated with Willingness to Share HGD Among Smartwatch Owners (n = 168).

Variable	aOR	95% CI	*p*-Value
**Smartwatch usage frequency**			
Non-user (ref)	1.00 (ref)	-	-
Low use (≤2/wk)	1.30	0.52–3.25	0.577
High use (≥3/wk)	1.64	0.77–3.49	0.203
**Trust in healthcare provider**			
Low (ref)	1.00 (ref)	-	-
Medium	0.95	0.23–3.95	0.943
High	1.77	0.42–7.51	0.441
**Age group**			
18–34 years (ref)	1.00 (ref)	-	-
35–49 years	0.89	0.41–1.95	0.775
50+ years	0.98	0.36–2.71	0.976
**Education level**			
Low (ref)	1.00 (ref)	-	-
Medium	1.33	0.37–4.80	0.667
High	1.34	0.52–3.43	0.547
**Health confidence**			
Low (ref)	1.00 (ref)	-	-
Moderate	1.35	0.23–7.94	0.743
High	1.33	0.23–7.55	0.747
**Regular health provider**			
No (ref)	1.00 (ref)	-	-
Yes	1.51	0.68–3.37	0.313

aOR: adjusted odds ratio; CI: confidence interval. Outcome: willingness to share health-generated data (104 willing, 64 not willing). likelihood-ratio χ^2^(11) = 8.03, *p* = 0.710; McFadden pseudo-R^2^ = 0.036; Nagelkerke R^2^ = 0.064; area under the ROC curve = 0.628; Hosmer–Lemeshow χ^2^(8) = 7.39, *p* = 0.495; mean variance inflation factor = 3.16.

**Table 5:** Bivariate Associations Between Participant Characteristics and Willingness to Share Health-Generated Data Among Owners (n = 168).

Variable	Willing (n = 104) n (%)	Not Willing (n = 64) n (%)	Pearson χ^2^	df	*p* (χ^2^)	Unadjusted OR (95% CI)
**Gender**						
Male (ref)	46 (44)	31 (48)	0.28	1	0.595	1.00 (ref)
Female	58 (56)	33 (52)				1.18 (0.63–2.21)
**Age group**						
18–34 (ref)	55 (53)	36 (56)	0.18	2	0.913	1.00 (ref)
35–49	33 (32)	19 (30)				1.14 (0.56–2.30)
50+	16 (15)	9 (14)				1.16 (0.46–2.92)
**Region**						
Central (ref)	92 (89)	62 (97)	3.67	1	0.055	1.00 (ref)
Other	12 (11)	2 (3)				4.04 (0.87–18.70)
**Education level**						
Low (ref)	17 (16)	12 (19)	0.18	2	0.914	1.00 (ref)
Medium	11 (11)	7 (11)				1.11 (0.33–3.69)
High	76 (73)	45 (70)				1.19 (0.52–2.72)
**Family monthly income**						
Low (ref)	11 (11)	10 (16)	1.04	2	0.593	1.00 (ref)
Medium	51 (49)	28 (44)				1.66 (0.63–4.38)
High	42 (40)	26 (41)				1.47 (0.55–3.94)
**Financial status**						
Difficult/very difficult (ref)	7 (7)	8 (12)	2.25	2	0.324	1.00 (ref)
Managing	42 (40)	28 (44)				1.71 (0.56–5.26)
Comfortable	55 (53)	28 (44)				2.24 (0.74–6.82)
**General health**						
Poor	3 (3)	0 (0)			0.266 *	Not estimable ^a^
Good (ref)	55 (53)	40 (63)				1.00 (ref)
Excellent	46 (44)	24 (37)				1.39 (0.74–2.64)
**Health confidence**						
Low (ref)	3 (3)	3 (5)			0.799 *	1.00 (ref)
Moderate	33 (32)	21 (33)				1.57 (0.29–8.53)
High	68 (65)	40 (62)				1.70 (0.33–8.83)
**Diabetes**						
No (ref)	97 (93)	59 (92)			0.768 *	1.00 (ref)
Yes	7 (7)	5 (8)				0.85 (0.26–2.81)
**Heart disease**						
No (ref)	99 (95)	62 (97)			0.710 *	1.00 (ref)
Yes	5 (5)	2 (3)				1.57 (0.29–8.32)
**Lung disease**						
No (ref)	101 (97)	64 (100)			0.288 *	1.00 (ref)
Yes	3 (3)	0 (0.0)				Not estimable
**Depression or anxiety**						
No (ref)	92 (89)	58 (91)	0.19	1	0.660	1.00 (ref)
Yes	12 (11)	6 (9)				1.26 (0.45–3.54)
**Weekly physical activity**						
<150 min/wk (ref)	63 (61)	38 (59)	0.02	1	0.877	1.00 (ref)
≥150 min/wk	41 (39)	26 (41)				0.95 (0.50–1.80)
**Regular health provider**						
No (ref)	73 (70)	52 (81)	2.54	1	0.111	1.00 (ref)
Yes	31 (30)	12 (19)				1.84 (0.86–3.92)
**Trust in family and friends**						
Low (ref)	31 (30)	26 (41)	2.07	2	0.355	1.00 (ref)
Medium	58 (56)	30 (47)				1.62 (0.82–3.21)
High	15 (14)	8 (12)				1.57 (0.58–4.29)
**Trust in healthcare provider**						
Low (ref)	5 (5)	4 (6)			0.114 *	1.00 (ref)
Medium	40 (38)	34 (53)				0.94 (0.23–3.79)
High	59 (57)	26 (41)				1.82 (0.45–7.31)
**Smartwatch usage frequency**						
Non-user (ref)	25 (24)	22 (34)	2.48	2	0.289	1.00 (ref)
Low use (≤2/wk)	22 (21)	14 (22)				1.38 (0.57–3.34)
High use (≥3/wk)	57 (55)	28 (44)				1.79 (0.86–3.72)
**Use of devices to discuss health**						
No (ref)	33 (32)	27 (42)	1.89	1	0.170	1.00 (ref)
Yes	71 (68)	37 (58)				1.57 (0.82–2.99)
**Sharing health-related information on social media**						
No (ref)	66 (64)	48 (75)	2.42	1	0.120	1.00 (ref)
Yes	38 (36)	16 (25)				1.73 (0.86–3.45)
**Use of online support groups**						
No (ref)	78 (75)	60 (94)	9.50	1	0.002	1.00 (ref)
Yes	26 (25)	4 (6)				5.00 (1.66–15.10)

OR: odds ratio; CI: confidence interval. Percentages are column percentages. * Fisher–Freeman–Halton Exact Test is reported instead of the Pearson χ^2^ for all variables with any expected cell counts below five. ^a^ Complete separation: all three owners reporting poor general health were willing to share, so the odds ratio for this category is not estimable.

## Data Availability

The data presented in this study are available from the corresponding author upon reasonable request.

## Abbreviations

The following abbreviations are used in this manuscript:

HINTS	Health Information National Trends Survey
HGD	Health-Generated Data

## Appendix A. Survey Used in This Study

Questionnaire for Study: Willingness to Share Smartwatch-Generated Health Data Among a Sample of Adults in Riyadh, Saudi Arabia.

### Appendix A.1. Sociodemographic Characteristics

What is your gender?	- Female - Male
What is your age group?	- 18–34 years - 35–49 years - 50–64 years - 65 years or older
What region in Saudi do you live in?	- Central - Northern - Southern - Eastern - Western
What is the highest level of education you have completed?	- High school graduate or less - Technical, vocational, or some college - College graduate - Postgraduate - Diploma
What is your total monthly household income?	- Less than 10,000 SAR - 10,000–20,000 SAR - 20,000–30,000 SAR - 30,000–40,000 SAR - Greater than 40,000 but below 100,000 SAR - 100,000 SAR and above
How would you describe your financial situation?	- Living comfortably - Managing with existing income - Difficult - Very difficult

### Appendix A.2. Health-Related Characteristics

In general, would you say your health is…	- Excellent - Good - Poor
How confident are you in your ability to take good care of your health?	- Completely confident - Very confident - Somewhat confident - A little confident - Not confident at all
Do you have any of the following chronic conditions?	- Diabetes - Heart Disease - Lung Disease - Depression or Anxiety - Cancer (excluding nonmelanoma skin cancer) - None of the above
On the days you do physical activity or exercise of at least moderate intensity, how long do you typically do these activities?	- Less than 150 min per week - 150 min or more per week
Do you have a regular doctor, nurse, or health professional that you see most often?	- Yes - No
How much do you trust information about health or medical topics from a doctor?	- Low - Medium - High
How much do you trust information about health or medical topics from family or friends?	- Low - Medium - High

### Appendix A.3

#### Appendix A.3.1. Part 1: Digital Health Behaviors

In the past 12 months, have you used the Internet for any of the following reasons? To share health information on social networking sites, such as Facebook or Twitter?	- Yes - No
In the past 12 months, have you used the Internet for any of the following reasons? To participate in an online forum or support group for people with a similar health or medical issue?	- Yes - No
Has your tablet or smartphone helped you in discussions with your health care provider?	- Yes - No
Do you own any wearable health devices?	- Yes - No

#### Appendix A.3.2. Part 2: Digital Health Behaviors

In the past month, how often did you use a wearable device to track your health	- almost every day - every day - 1–2 times per week - less than once per week - did not use a wearable device in the past month
Will you be willing to share your wearable data?	- Yes - No

### Appendix A.4. Willingness to Share Health Data

Would you be willing to share health data from your wearable device with your healthcare provider?	- Yes - No
Would you be willing to share health data from your wearable device with your family or friends?	- Yes - No

## Appendix B

**Table A1:** Multivariable Logistic Regression of Factors Associated with Smartwatch Ownership (n = 391).

Variable	aOR	95% CI	*p*-Value
**Gender**			
Male (ref)	1.00 (ref)	-	-
Female	0.75	0.47–1.21	0.241
**Age group**			
18–34 years (ref)	1.00 (ref)	-	-
35–49 years	0.82	0.47–1.41	0.468
50+ years	0.20	0.11–0.39	<0.001
**Education level**			
Low (ref)	1.00 (ref)	-	-
Medium	1.78	0.72–4.44	0.212
High	1.37	0.72–2.59	0.335
**Monthly income**			
Low (ref)	1.00 (ref)	-	-
Medium	0.84	0.39–1.82	0.665
High	1.47	0.63–3.40	0.370
**Financial status**			
Difficult or very difficult (ref)	1.00 (ref)	-	-
Managing with existing income	0.95	0.41–2.21	0.898
Living comfortably	1.28	0.53–3.08	0.589
**General health**			
Poor (ref)	1.00 (ref)	-	-
Good	4.63	1.09–19.65	0.038
Excellent	4.63	1.02–20.95	0.046
**Health confidence**			
Low (ref)	1.00 (ref)	-	-
Moderate	1.47	0.51–4.23	0.473
High	2.19	0.76–6.33	0.146
**Any chronic condition**			
No (ref)	1.00 (ref)	-	-
Yes	1.88	1.03–3.42	0.039
**Weekly physical activity**			
Less than 150 min per week (ref)	1.00 (ref)	-	-
150 min or more per week	1.47	0.90–2.40	0.123
**Regular health provider**			
No (ref)	1.00 (ref)	-	-
Yes	1.45	0.82–2.58	0.206
**Trust in family and friends**			
Low (ref)	1.00 (ref)	-	-
Medium	1.31	0.79–2.17	0.292
High	0.99	0.46–2.11	0.982
**Trust in healthcare provider**			
Low (ref)	1.00 (ref)	-	-
Medium	0.65	0.22–1.90	0.427
High	0.61	0.20–1.84	0.378

aOR: adjusted odds ratio; CI: confidence interval. Outcome: smartwatch ownership (168 owners, 223 non-owners). Model fit: likelihood-ratio χ^2^(20) = 64.33, *p* < 0.001; McFadden pseudo-R^2^ = 0.120; Nagelkerke R^2^ = 0.204; area under the ROC curve = 0.723; Hosmer–Lemeshow χ^2^(8) = 6.18, *p* = 0.627; mean variance inflation factor = 3.27. The wide confidence intervals for general health reflect the small number of participants reporting poor health (n = 17) and these estimates should be interpreted with caution.

## References

[1] Casselman J., Onopa N., Khansa L. (2017). Wearable healthcare: Lessons from the past and a peek into the future. Telemat. Inform..

[2] Aminabee S. (2024). The Future of Healthcare and Patient-Centric Care: Digital Innovations, Trends, and Predictions. Emerging Technologies for Health Literacy and Medical Practice.

[3] Ghadi Y.Y., Shah S.F.A., Waheed W., Mazhar T., Ahmad W., Saeed M.M., Hamam H. (2025). Integration of wearable technology and artificial intelligence in digital health for remote patient care. J. Cloud Comput..

[4] Walker D.M., Sieck C.J., Menser T., Huerta T.R., Scheck McAlearney A. (2017). Information technology to support patient engagement: Where do we stand and where can we go?. J. Am. Med. Inform. Assoc..

[5] Godfrey A., Hetherington V., Shum H., Bonato P., Lovell N.H., Stuart S. (2018). From A to Z: Wearable technology explained. Maturitas.

[6] Giugliano G., Capece S., Laudante E., Martinez Muñoz V.F., Buono M. (2026). Smart Wearable Devices and Technologies for Human-Augmentation Industry 5.0: A Systematic Review. Appl. Sci..

[7] Escobar-Linero E., Muñoz-Saavedra L., Luna-Perejón F., Sevillano J.L., Domínguez-Morales M. (2023). Wearable Health Devices for Diagnosis Support: Evolution and Future Tendencies. Sensors.

[8] Köhler C., Bartschke A., Fürstenau D., Schaaf T., Salgado-Baez E. (2024). The Value of Smartwatches in the Health Care Sector for Monitoring, Nudging, and Predicting: Viewpoint on 25 Years of Research. J. Med. Internet Res..

[9] Tung F.W. (2026). Self-quantification and Data Engagement: Insights from Smartwatch Users. HCI International 2025—Late Breaking Papers.

[10] Petek B.J., Al-Alusi M.A., Moulson N., Grant A.J., Besson C., Guseh J.S., Wasfy M.M., Gremeaux V., Churchill T.W., Baggish A.L. (2023). Consumer Wearable Health and Fitness Technology in Cardiovascular Medicine: JACC State-of-the-Art Review. J. Am. Coll. Cardiol..

[11] Phillips S.M., Cadmus-Bertram L., Rosenberg D., Buman M.P., Lynch B.M. (2018). Wearable Technology and Physical Activity in Chronic Disease: Opportunities and Challenges. Am. J. Prev. Med..

[12] Murala D.K., Panda S.K., Dash S.P. (2023). MedMetaverse: Medical Care of Chronic Disease Patients and Managing Data Using Artificial Intelligence, Blockchain, and Wearable Devices State-of-the-Art Methodology. IEEE Access.

[13] Mattison G., Canfell O., Forrester D., Dobbins C., Smith D., Töyräs J., Sullivan C. (2022). The Influence of Wearables on Health Care Outcomes in Chronic Disease: Systematic Review. J. Med. Internet Res..

[14] Kamei T., Kanamori T., Yamamoto Y., Edirippulige S. (2022). The use of wearable devices in chronic disease management to enhance adherence and improve telehealth outcomes: A systematic review and meta-analysis. J. Telemed. Telecare.

[15] Pal D., Funilkul S., Vanijja V. (2020). The future of smartwatches: Assessing the end-users’ continuous usage using an extended expectation-confirmation model. Univers. Access Inf. Soc..

[16] Alanazi A., Alanazi M., Aldosari B. (2023). Personal Health Record (PHR) Experience and Recommendations for a Transformation in Saudi Arabia. J. Pers. Med..

[17] Simpson E., Brown R., Sillence E., Coventry L., Lloyd K., Gibbs J., Tariq S., Durrant A.C. (2021). Understanding the Barriers and Facilitators to Sharing Patient-Generated Health Data Using Digital Technology for People Living with Long-Term Health Conditions: A Narrative Review. Front. Public Health.

[18] Park Y.E., Beck S.S., Lee Y. (2025). Perceptions and Willingness of Patients and Caregivers on the Utilization of Patient-Generated Health Data: A Cross-Sectional Survey. Int. J. Environ. Res. Public Health.

[19] Seltzer E., Goldshear J., Guntuku S.C., Grande D., Asch D.A., Klinger E.V., Merchant R.M. (2019). Patients’ willingness to share digital health and non-health data for research: A cross-sectional study. BMC Med. Inf. Decis. Mak..

[20] Baines R., Stevens S., Austin D., Anil K., Bradwell H., Cooper L., Maramba I.D., Chatterjee A., Leigh S. (2024). Patient and Public Willingness to Share Personal Health Data for Third-Party or Secondary Uses: Systematic Review. J. Med. Internet Res..

[21] Rising C.J., Gaysynsky A., Blake K.D., Jensen R.E., Oh A. (2021). Willingness to Share Data from Wearable Health and Activity Trackers: Analysis of the 2019 Health Information National Trends Survey Data. JMIR mHealth uHealth.

[22] Chandrasekaran R., Muhammed Sadiq T., Moustakas E. (2025). Usage Trends and Data Sharing Practices of Healthcare Wearable Devices Among US Adults: Cross-Sectional Study. J. Med. Internet Res..

[23] Khatiwada P., Yang B., Lin J.C., Blobel B. (2024). Patient-Generated Health Data (PGHD): Understanding, Requirements, Challenges, and Existing Techniques for Data Security and Privacy. J. Pers. Med..

[24] Faridoon A., Kechadi M.T. (2024). Healthcare Data Governance, Privacy, and Security—A Conceptual Framework. Body Area Networks: Smart IoT and Big Data for Intelligent Health Management (BodyNets 2024).

[25] Rosenbaum S. (2010). Data Governance and Stewardship: Designing Data Stewardship Entities and Advancing Data Access. Health Serv. Res..

[26] Zhang L., Xie Q., Qiu S., Liu M., You F., Zhao X. (2025). Willing or reluctant to share health data? A moderated mediation analysis of wearable device usage and data-sharing intentions among older adults. Digit. Health.

[27] Alaskari E., Alanzi T., Alrayes S., Aljabri D., Almulla S., Alsalman D., Algumzi A., Alameri R., Alakrawi Z., Alnaim N. (2022). Utilization of wearable smartwatch and its application among Saudi population. Front. Comput. Sci..

[28] Green G., Soleton M., Hokroh M. (2020). Factors Influencing Health Wearables Adoption and Usage in Saudi Arabia. Turk. Tur. Arastirmalari Derg..

[29] Health Sector Transformation Program. Vision Relization Program. https://www.vision2030.gov.sa/en/explore/programs/health-sector-transformation-program.

[30] National Transformation Program|Saudi Vision 2030. https://www.vision2030.gov.sa/en.

[31] Mani Z.A., Goniewicz K. (2024). Transforming Healthcare in Saudi Arabia: A Comprehensive Evaluation of Vision 2030’s Impact. Sustainability.

[32] Riaz H., Naqvi R.A., Ellahi M., Usman M.A., Usman M.R., Jeong D., Lee S.W. (2026). Robust Steganography Technique for Enhancing the Protection of Medical Records in Healthcare Informatics. IEEE J. Biomed. Health Inform..

[33] Suleiman A.K., Ming L.C. (2025). Transforming healthcare: Saudi Arabia’s vision 2030 healthcare model. J. Pharm. Policy Pract..

[34] HINTS Instrument Development and Validation Procedures. https://hints.cancer.gov/about-hints/instrument-development.aspx.

[35] All HINTS Questions. https://hints.cancer.gov/view-questions/all-hints-questions.aspx.

[36] Sousa V.D., Rojjanasrirat W. (2011). Translation, adaptation and validation of instruments or scales for use in cross-cultural health care research: A clear and user-friendly guideline. J. Eval. Clin. Pract..

[37] Beaton D.E., Bombardier C., Guillemin F., Ferraz M.B. (2000). Guidelines for the process of cross-cultural adaptation of self-report measures. Spine.

[38] Muhib F.B., Lin L.S., Stueve A., Miller R.L., Ford W.L., Johnson W.D., Smith P.J. (2001). A venue-based method for sampling hard-to-reach populations. Public Health Rep..

[39] Aldekhyyel R.N., Alshuaibi F., Alsaaid O., Bin Moammar F., Alanazy T., Namshah A., Altassan K., Aldekhyyel R., Jamal A. (2024). Exploring behavioral intention to use telemedicine services post COVID-19: A cross sectional study in Saudi Arabia. Front. Public Health.

[40] Balkhy H.H., Abolfotouh M.A., Al-Hathlool R.H., Al-Jumah M.A. (2010). Awareness, attitudes, and practices related to the swine influenza pandemic among the Saudi public. BMC Infect. Dis..

[41] Abdulrashid O.A., Shah H.B.U., Baeshen W.A., Aljuaid S.M., Alasmari E.A., Baokbah R.A., Alamoudi N.M., Alkhelewi M.S., Turkistani A.A., Alharbi A.A. (2023). Physical activity and health-related quality of life among adults living in Jeddah city Saudi Arabia. PeerJ.

[42] Roos L.G., Slavich G.M. (2023). Wearable technologies for health research: Opportunities, limitations, and practical and conceptual considerations. Brain Behav. Immun..

[43] Vaidyam A., Halamka J., Torous J. (2022). Enabling Research and Clinical Use of Patient-Generated Health Data (the mindLAMP Platform): Digital Phenotyping Study. JMIR mHhealth uHealth.

[44] Yen H.Y., Liao Y., Huang H.Y. (2022). Smart Wearable Device Users’ Behavior Is Essential for Physical Activity Improvement. Int. J. Behav. Med..

[45] O’Connor S., Hanlon P., O’Donnell C.A., Garcia S., Glanville J., Mair F.S. (2016). Understanding factors affecting patient and public engagement and recruitment to digital health interventions: A systematic review of qualitative studies. BMC Med. Inf. Decis. Mak..

[46] Canali S., Schiaffonati V., Aliverti A. (2022). Challenges and recommendations for wearable devices in digital health: Data quality, interoperability, health equity, fairness. PLoS Digit. Health.

